# Supplementary material for: Fabrication of Metallic Superhydrophobic Surfaces with Tunable Condensate Self-Removal Capability and Excellent Anti-Frosting Performance
Source: Nanomaterials (Basel). 2022 Oct 18;12(20):3655. doi: 10.3390/nano12203655 (PMC9611512; doi:10.3390/nano12203655)
Supplement: Supplementary file 1 [file nanomaterials-12-03655-s001.zip › nanomaterials-1930985-Supplementary materials.pdf]

nanomaterials

Supplementary Materials

Fabrication of Metallic Superhydrophobic Surfaces with Tunable Condensate Self-Removal Capability and Excellent Anti-Frosting Performance

Jian-Guo He [1,2,3], Guan-Lei Zhao [4,5,*], Shou-Jun Dai [1,3], Ming Li [6], Gui-Sheng Zou [5], Jian-Jun Wang [7], Yang Liu [1,3], Jia-Qi Yu [1,3], Liang-Fei Xu [4], Jian-Qiu Li [4], Lian-Wen Fan [8] and Min Huang [1,3,*]

1 Aerospace Information Research Institute, Chinese Academy of Sciences, Beijing 100094, China; hejg@aircas.ac.cn (J.-G.H.); daishoujun@aoe.ac.cn (S.-J.D.); liuyang@aircas.ac.cn (Y.L.); yujq@aircas.ac.cn (J.-Q.Y.)
2 School of Optoelectronics, University of Chinese Academy of Sciences, Beijing 100049, China
3 Key Laboratory of Computational Optical Imaging Technology, Chinese Academy of Sciences, Beijing 100094, China
4 State Key Laboratory of Automotive Safety and Energy, School of Vehicle and Mobility, Tsinghua University, Beijing 100084, China; xuliangfei@tsinghua.edu.cn (L.-F.X.); lijianqiu@tsinghua.edu.cn (J.-Q.L.)
5 State Key Laboratory of Tribology, Key Laboratory for Advanced Manufacturing by Materials Processing Technology, Ministry of Education of PR China, Department of Mechanical Engineering,Tsinghua University, Beijing 100084, China; zougsh@tsinghua.edu.cn (G.-S.Z.)
6 State Key Laboratory of Transient Optics and Photonics, Xi'an Institute of Optics and Precision Mechanics of CAS, Xi'an 710119, China; liming@opt.ac.cn (M.L.)
7 Institute of Chemistry, Chinese Academy of Sciences, Beijing 100190, China; wangj220@iccas.ac.cn (J.-J.W.)
8 Technology and Engineering Center for Space Utilization, Chinese Academy of Sciences, Beijing 100094, China; fanlw@csu.ac.cn (L.-W.F.)
* Correspondence: zgl1989@mail.tsinghua.edu.cn (G.-L.Z.); huangmin@aircas.ac.cn (M.H.)

Citation: He, J.-G.; Zhao, G.-L.; Dai, S.-J.; Li, M.; Zou, G.-S.; Wang, J.-J.; Liu, Y.; Yu, J.-Q.; Xu, L.-F.; Li, J.-Q.; et al. Fabrication of Metallic Superhydrophobic Surfaces with Tunable Condensate Self-Removal Capability and Excellent Anti-Frosting Performance. *Nanomaterials* **2022**, *12*, 3655. https://doi.org/10.3390/nano12203655

Academic Editors: Yang-Tse Cheng, Ion N. Mihailescu

Received: 7 September 2022
Accepted: 14 October 2022
Published: 18 October 2022

Publisher's Note: MDPI stays neutral with regard to jurisdictional claims in published maps and institutional affiliations.

Copyright: © 2022 by the authors. Licensee MDPI, Basel, Switzerland. This article is an open access article distributed under the terms and conditions of the Creative Commons Attribution (CC BY) license (https://creativecommons.org/licenses/by/4.0/).

Supporting Information: Videos:

Video S1. Top view of inefficient self-propelled droplet jumping on S2-Cu.
Video S2. Top view of efficient self-propelled droplet jumping on S3-Cu.
Video S3. Side view of self-propelled droplet jumping on S3-Cu.
Video S4. Top view of efficient self-propelled droplet jumping on S3-Al.
Video S5. Top view of efficient self-propelled droplet jumping on S3-Steel.
Video S6. Top view of efficient self-propelled droplet jumping on S3-Ti.

Supporting Information: Tables

Table S1. List of the prepared samples and their respective laser processing parameters.

Copper alloy sample #	Aluminum sample #	Carbon steel sample #	Titanium alloy sample #	Fluence (J/cm²)	PRF (kHz)
Cu-1	Al-1	Steel-1	Ti-1	0.032	2000
Cu-2	Al-2	Steel-2	Ti-2	0.043	1500
Cu-3	Al-3	Steel-3	Ti-3	0.063	1000
Cu-4	Al-4	Steel-4	Ti-4	0.103	600
Cu-5	Al-5	Steel-5	Ti-5	0.194	300
Cu-6	Al-6	Steel-6	Ti-6	0.16	2000
Cu-7	Al-7	Steel-7	Ti-7	0.211	1500
Cu-8	Al-8	Steel-8	Ti-8	0.313	1000
Cu-9	Al-9	Steel-9	Ti-9	0.507	600
Cu-10	Al-10	Steel-10	Ti-10	0.96	300
Cu-11	Al-11	Steel-11	Ti-11	0.362	2000
Cu-12	Al-12	Steel-12	Ti-12	0.478	1500
Cu-13	Al-13	Steel-13	Ti-13	0.711	1000
Cu-14	Al-14	Steel-14	Ti-14	1.151	600
Cu-15	Al-15	Steel-15	Ti-15	2.182	300
Cu-16	Al-16	Steel-16	Ti-16	0.896	2000
Cu-17	Al-17	Steel-17	Ti-17	1.183	1500
Cu-18	Al-18	Steel-18	Ti-18	1.759	1000
Cu-19	Al-19	Steel-19	Ti-19	2.838	600
Cu-20	Al-20	Steel-20	Ti-20	5.437	300
Cu-21	Al-21	Steel-21	Ti-21	1.786	2000
Cu-22	Al-22	Steel-22	Ti-22	2.36	1500
Cu-23	Al-23	Steel-23	Ti-23	3.507	1000
Cu-24	Al-24	Steel-24	Ti-24	5.683	600
Cu-25	Al-25	Steel-25	Ti-25	10.754	300

Table S2. Summary of contact angle analysis for Cu samples.

Cu	Advancing CA (°)	Receding CA (°)
**S1-Cu**	145.5±1.5	N/A
**S2-Cu**	146.8±1.6	134.8±3.7
**S3-Cu**	163.4±2.1	159.3±2.2

Table S3. Summary of contact angle analysis for Al, Steel, and Ti samples.

Al	Advancing CA (°)	Receding CA (°)
**S1-Al**	143.1±1.2	N/A
**S2-Al**	145.3±0.9	108±4.9
**S3-Al**	160.3±0.6	154.0±1.6

**Steel**		
**S1-Steel**	142.1±1.6	N/A
**S2-Steel**	144.5±1.8	123.4±4.4
**S3-Steel**	156.3±1.4	148.2±1.9
**Ti**		
**S1-Ti**	142.1±1.6	N/A
**S2-Ti**	146.6±1.4	127.8±2
**S3-Ti**	160.2±1.8	152.5±1.8

Supporting Information: Figures

S1. Surface Fabrication and Characterization for Copper-Zinc Alloy

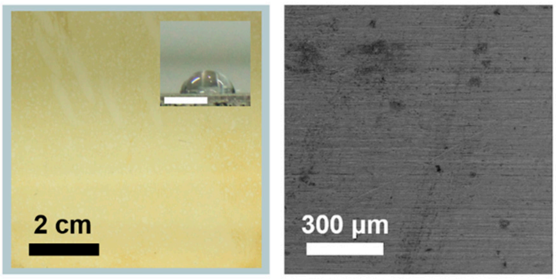

Figure S1. Bare Cu plate before laser processing and its SEM image, the scale bar in the inset is 2 mm.

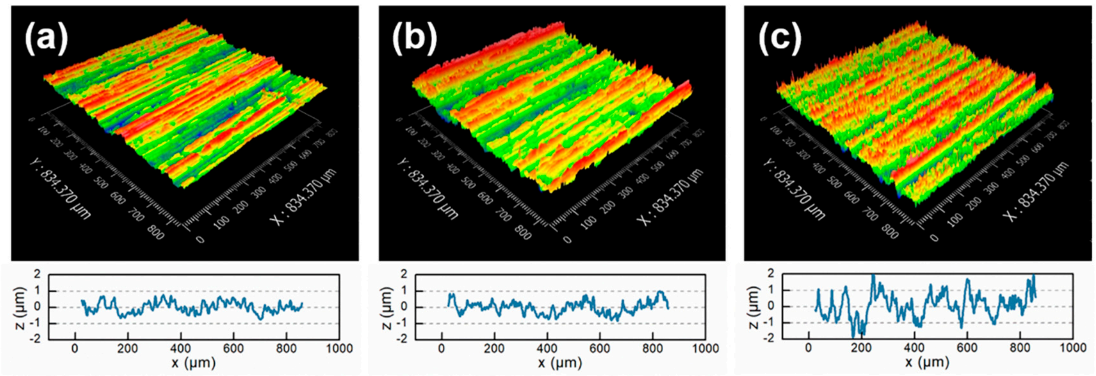

Figure S2. White light interferometry images of the ultrafast laser processed copper-zinc alloy surfaces for (**a**) S1-Cu, (**b**) S2-Cu, and (**c**) S3-Cu.

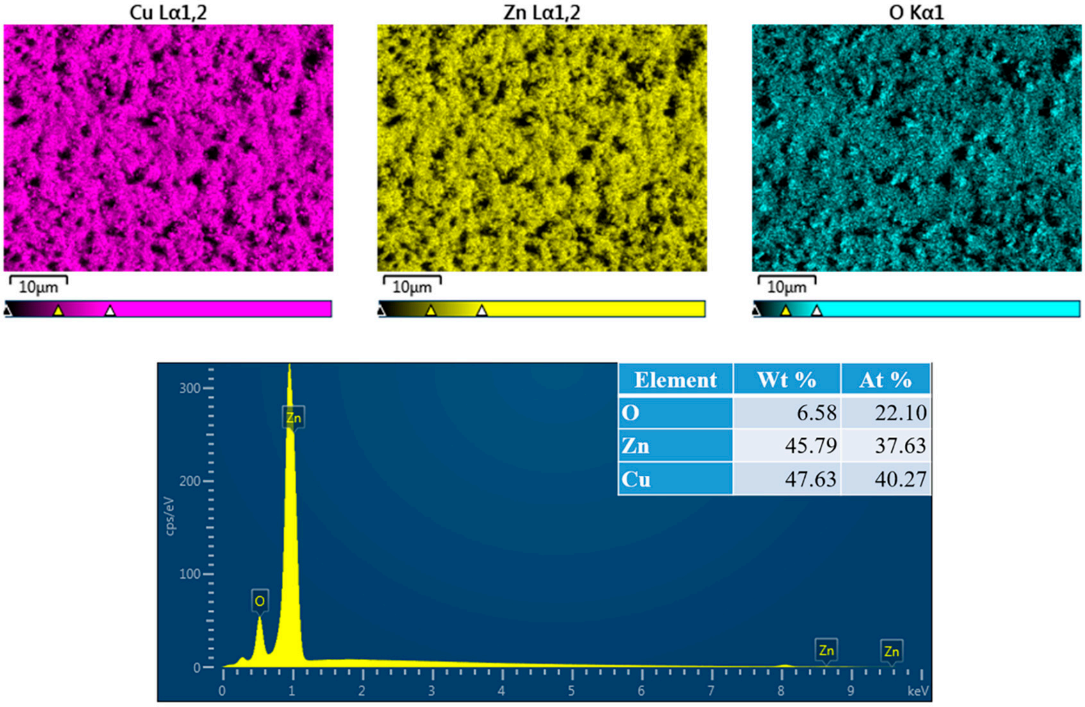

Figure S3. Energy-dispersive X-ray spectroscopy (EDS) analysis of ultrafast laser processed Cu plate, it is shown that the nanostructured Cu surface is oxidized.

S2. Characterization of Surface Wetting Properties

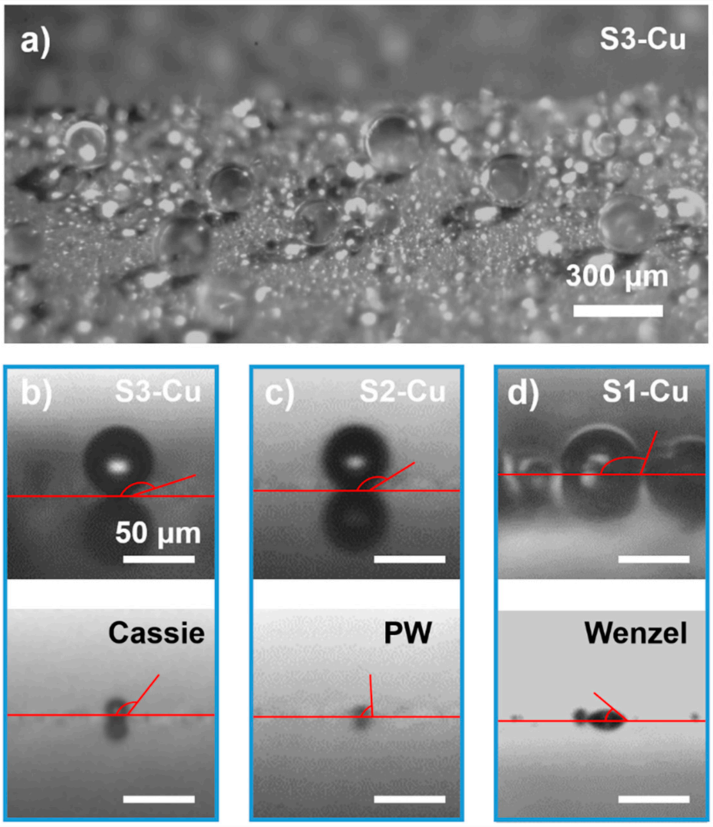

Figure S4. Wetting properties of condensed microdroplets on the prepared surfaces. (**a**) S3-Cu is superhydrophobic to condensed microdroplets. (**b–d**) Estimation of advancing and receding contact angle for (**b**) S3-Cu (165 ± 5°, 135 ± 10°), (**c**) S2-Cu (155 ± 5°, 90 ± 10°), and (**d**) S1-Cu (130 ± 5°, 30 ± 10°).

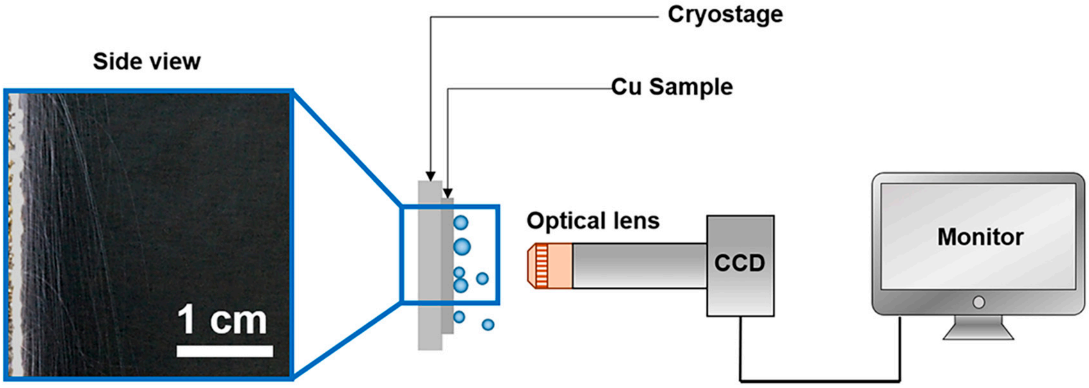

Figure S5. Schematic illustration of the setup for condensing experiments. Samples were bonded with thermal paste onto a cryostage vertically mounted in a chamber with an ambient temperature of 23.4 ± 2.5 °C. The temperature of the cryostage and humidity were accurately controlled to be 1 ± 1.0 °C and 55.8 ± 5%, respectively. A high-speed video camera mounted with optical lens was used to record the condensing process.

S3. Effect of Wetting Property on Coalesced Droplet Jumping Capability

According to Boreyko et al., the initial velocity of the coalescence-induced jumping droplet can be calculated as:[1]

$$v = \sqrt{\frac{2E_k}{m}}, \quad \text{(S1)}$$

where E_k is the kinetic energy responsible for the off-surface motion of the coalesced droplet as:

$$E_k = \eta \Delta E_s - E_{ad}. \quad \text{(S2)}$$

where η is an initial droplet size mismatch (M) related dissipation factor, ΔE_s is the released surface energy between the initial state and the final state of the droplet coalescence process (Figure S6), and E_{ad} is the solid-liquid adhesion between the droplets and the surface.[2] ΔE_s can be obtained by calculating the difference in surface area between the initial ($E_{s,i}$) and the final ($E_{s,f}$) state of the droplets as:

$$\Delta E_s = E_{s,i} - E_{s,f}, \quad \text{(S3)}$$

For simple illustration, this analysis considers the coalescing of two droplets with the same radius (R_i), as shown in Figure S6. By considering $E_s = \sigma_{lv} A_{lv} + \sigma_{sl} A_{sl} + \sigma_{sv} A_{sv}$, ($\sigma$ and A are surface tension and interface area, respectively) and utilizing Young's relation ($\sigma_{sv} = \sigma_{lv} \cos\theta + \sigma_{sl}$), the released surface energy can be calculated as:

$$\Delta E_s = 2\sigma_{lv}(A_{i,lv} - A_{i,sl} \cos\theta_{static}) - \sigma_{lv} A_{f,lv}, \quad \text{(S4)}$$

where $A_{i,lv}$ represents the liquid-vapor interface area, $A_{i,sl}$ represents the solid-liquid interface area of the droplets in the initial state. $A_{f,lv}$ is the liquid-vapor interface area of the droplet in the final state. θ_{adv} is the apparent ACA of the droplets in the initial state.

In addition, the respective area terms can be calculated by considering droplets in the initial state as spherical caps, and droplet in the final state as a sphere:[3]

$$A_{i,lv} = 2\pi R_i^2 (1 - \cos\theta_{adv}) + (1 - \varphi)\pi R_i^2 \sin^2\theta_{adv}, \quad \text{(S5)}$$

$$A_{i,sl} = \varphi \pi R_i^2 \sin^2\theta_{adv}, \quad \text{(S6)}$$

$$A_{f,lv} = 4\pi R_f^2, \quad \text{(S7)}$$

$$R_f = \left(\frac{3V_i}{2\pi}\right)^{1/3}, \text{ and} \quad \text{(S8)}$$

$$V_i = \frac{\pi}{3} R_i^3 (2 + \cos\theta_{adv})(1 - \cos\theta_{adv})^2, \quad \text{(S9)}$$

where R_i and R_f are the radii of the droplets in the initial and the final state, respectively. V_i is the volume of the droplet in the initial state. φ is solid fraction between the droplets and the surface. Furthermore, it should be noted that for two droplets of equal size, η is around 5.6%.[2]

The adhesion E_{ad} can be calculate for two droplets system according to McCarthy et al. as:[4]

$$E_{ad} = 2A_{i,sl}\sigma_{lv}(1 + \cos\theta_{rec}), \quad \text{(S10)}$$

where θ_{rec} is the RCA of the droplets in the initial state. However, according to Seo et al., when the surface is partially wetting by condensed water, a surface partial-wetting term should be considered when analyzing adhesion.[5] Herein, ϕ_{pw} is defined as the degree of partial-wetting. For droplets in Wenzel state (Figure S6a), the surface structure is completed wetted by the condensed droplets ($\phi_{pw} = 1$), meaning the lower caps of the droplets

contact with both the tips of the nanostructures (liquid-solid), and liquid bridge within the surface structures (liquid-liquid). For droplets in CB state (Figure S6c), no wetting of the surface structure occurs ($\phi_{pw} = 0$), this implies that the lower caps of the droplets contact with both the tips of the nanostructures (liquid-solid) and the air gap (liquid-vapor). Interestingly, for droplets in partial-wetting (PW) state ($0 < \phi_{pw} < 1$), the lower caps of the condensed droplets exhibit all liquid-solid, liquid-vapor, and liquid-liquid contacts, as illustrated in Figure S6b. Therefore, by considering partial-wetting of the surface structure, and assuming the jumping droplet would break at the liquid-liquid interface leaving a wetted patch of water, the adhesion term ($E_{ad,pw}$) then becomes:[6]

$$E_{ad,pw} = 2A_{i,sl}\sigma_{lv}(1 + cos\,\theta_{rec}) + 2A_{ds}(1 - \varphi)\phi_{pw}\,(1 + cos\,\theta_w), \qquad (S11)$$

where $A_{ds} = \pi R_i^2 sin^2\theta_{adv}$ is the contact area between the droplet and the surface, and the θ_w (0°) is the RCA between the droplet and the liquid bridge within the nanostructures.

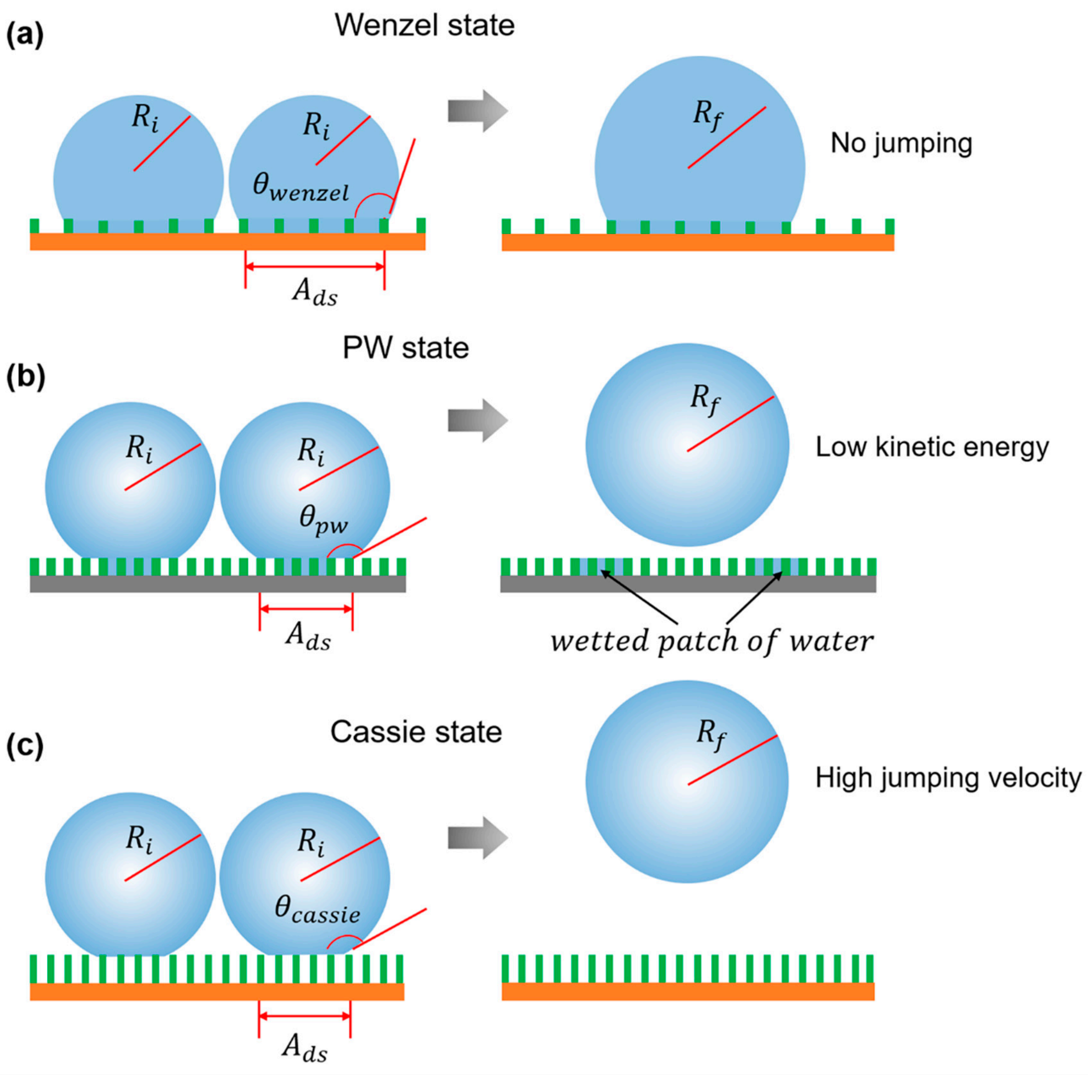

Figure S6. Schematic illustration of wetting states for the coalescing droplets on (**a**) S1-Cu (Wenzel), (**b**) S2-Cu (PW), and (**c**) S3-Cu (CB).

To more explicitly illustrate the effect of partial-wetting on droplet jumping capability. The jumping velocity of the coalesced droplet is plotted with respect to the radii of the coalescing droplets in different wetting states. As shown in Figure S7, the droplet jumping

capability reduces significantly with increase in degree of partial-wetting (0 for CB, and 1 for Wenzel).

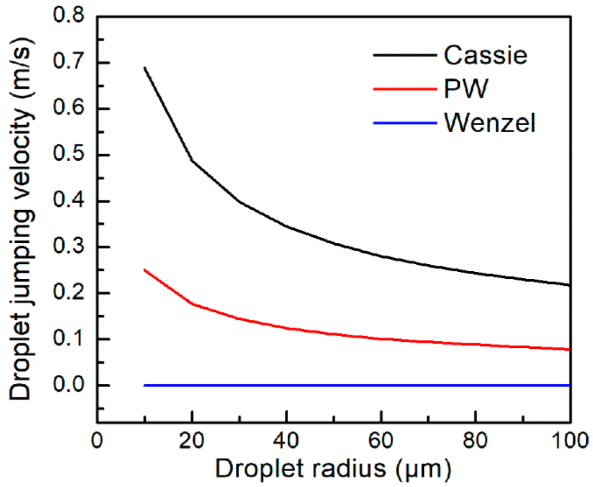

Figure S7. Droplet jumping velocity with respect to initial droplet radius (two droplets system with the same radius). The results obtained from Figure S4 were used as the values for theoretical calculation, and values for solid fraction are adopted from previous studies (ACA = 165 ± 5°, RCA = 135 ± 10°, $\varphi = 0.02$, and $\phi_{pw} = 0$ for S3-Cu, ACA = 155 ± 5°, RCA = 90 ± 10°, $\varphi = 0.02$, and $\phi_{pw} = 0.1$ for S2-Cu, and ACA = 130 ± 5°, RCA = 30 ± 10°, and $\phi_{pw} = 1$ for S1-Cu) [7].

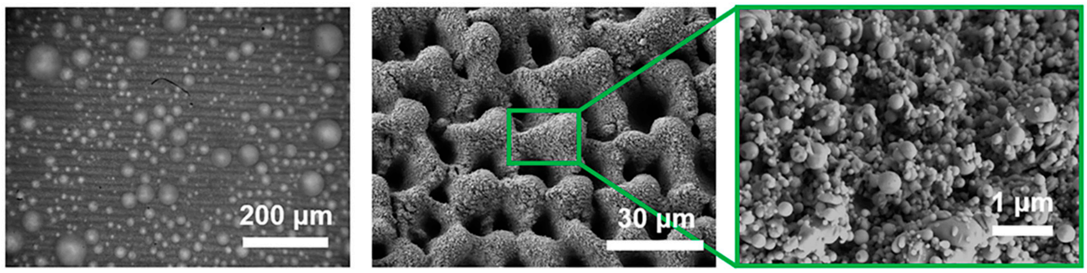

Figure S8. High laser fluence resulted in severe melting of the Cu surface due to insufficient heat dissipation. Droplet jumping frequency on this surface was comparable to S3-Cu, but the amount of accumulated water was increased due to more surface area for condensed droplets to nucleate and grow.

S4. Frosting Experiments and Results

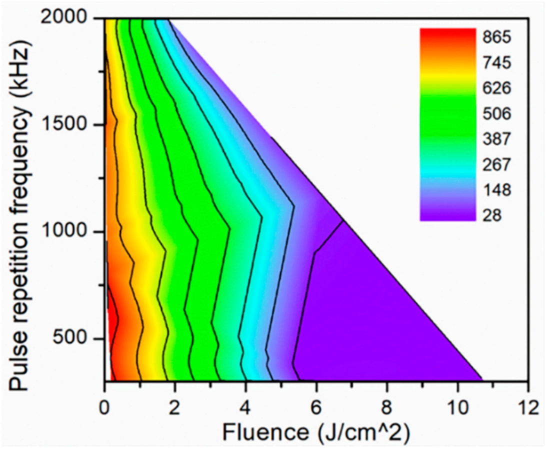

Figure S9. Phase map relating laser processing parameters with the number of accumulated large droplets for Cu (defined as droplet radius of over 50 μm). The red region represents the highest number of accumulated large droplets, and the purple region represents the lowest number accumulated large droplets. Unit for the number of accumulated large droplets is cm^{-2} over 30 minutes of condensing experiment.

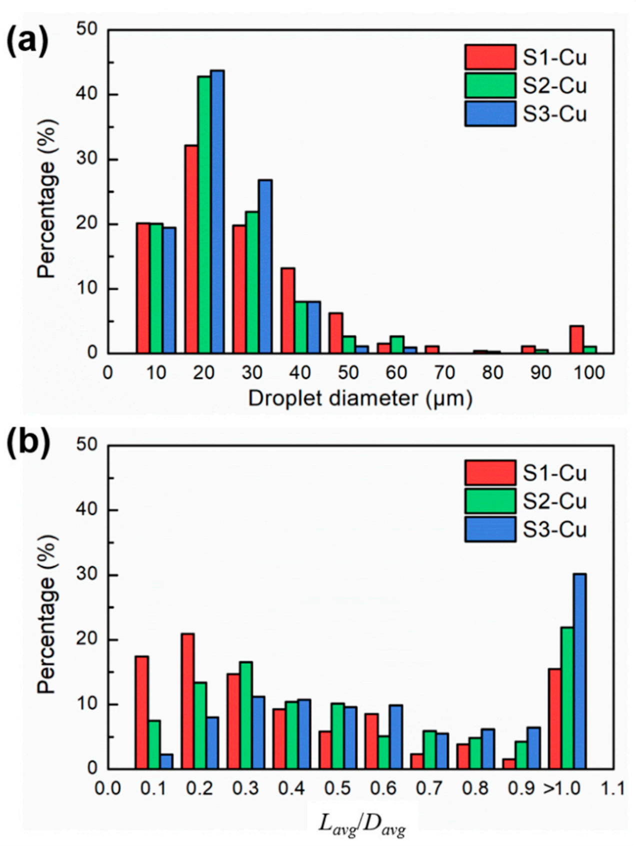

Figure S10. Histograms of the liquid droplet diameter (D_{avg}) and L_{avg}/D_{avg} during the frosting process on S1-Cu, S2-Cu and S3-Cu. (**a**) The average *D* are 26.33 μm, 20.22 μm, and 17.93 μm for S1-Cu, and S2-Cu, and S3-Cu, respectively. In addition, there were only a few of condensed droplets which were larger than 100 μm on S3-Cu, while 4.26% and 1.07% of the droplets were larger than 100 μm on S1-Cu and S2-Cu, which served as preferable ice nucleation sites. (**b**) On S3-Cu, more than 30% of droplets exhibited L_{avg}/D_{avg} >1, compared to 15.50% and 21.93% for S1-Cu and S2-Cu.

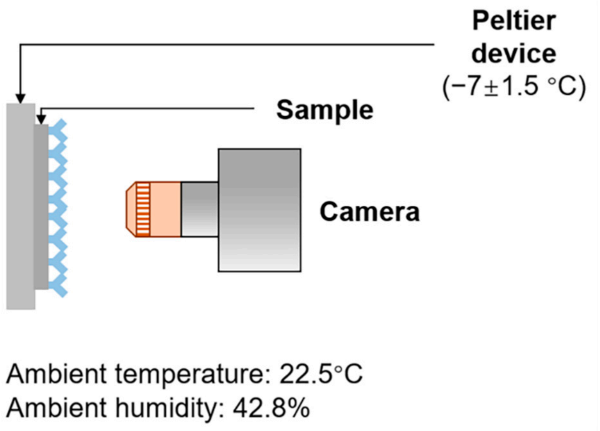

Figure S11. Frosting test setup. The samples were bonded onto a vertically oriented Peltier device with thermal paste. The ambient relative humidity and temperature were 42.8 ± 5% and 22.5 ± 2.5

°C, respectively. The cooling plate was held at a temperature of −7 ± 1.5 °C (*SSD* = 2.45), and the frosting processes of the samples were recorded with a digital camera (Canon EOS 60d).

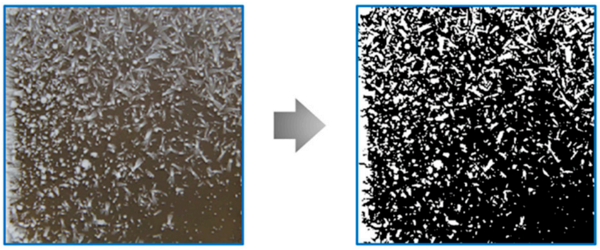

Figure S12. Frost coverage calculation using an image processing and pixel counting method. The original image from the frosting test was converted to binary image, and a computer algorithm was employed to calculate the fraction of frost (white pixels) against the background (black).

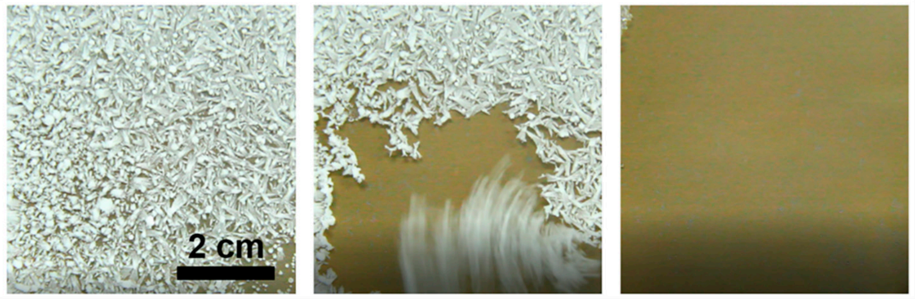

Figure S13. Defrosting on Cu-S3. It can be observed that when the substrate temperature increased to above 0°, frost layer quickly peeled off the surface without leaving any residue droplet.

S5. Material Adaptability of the Fabrication Method

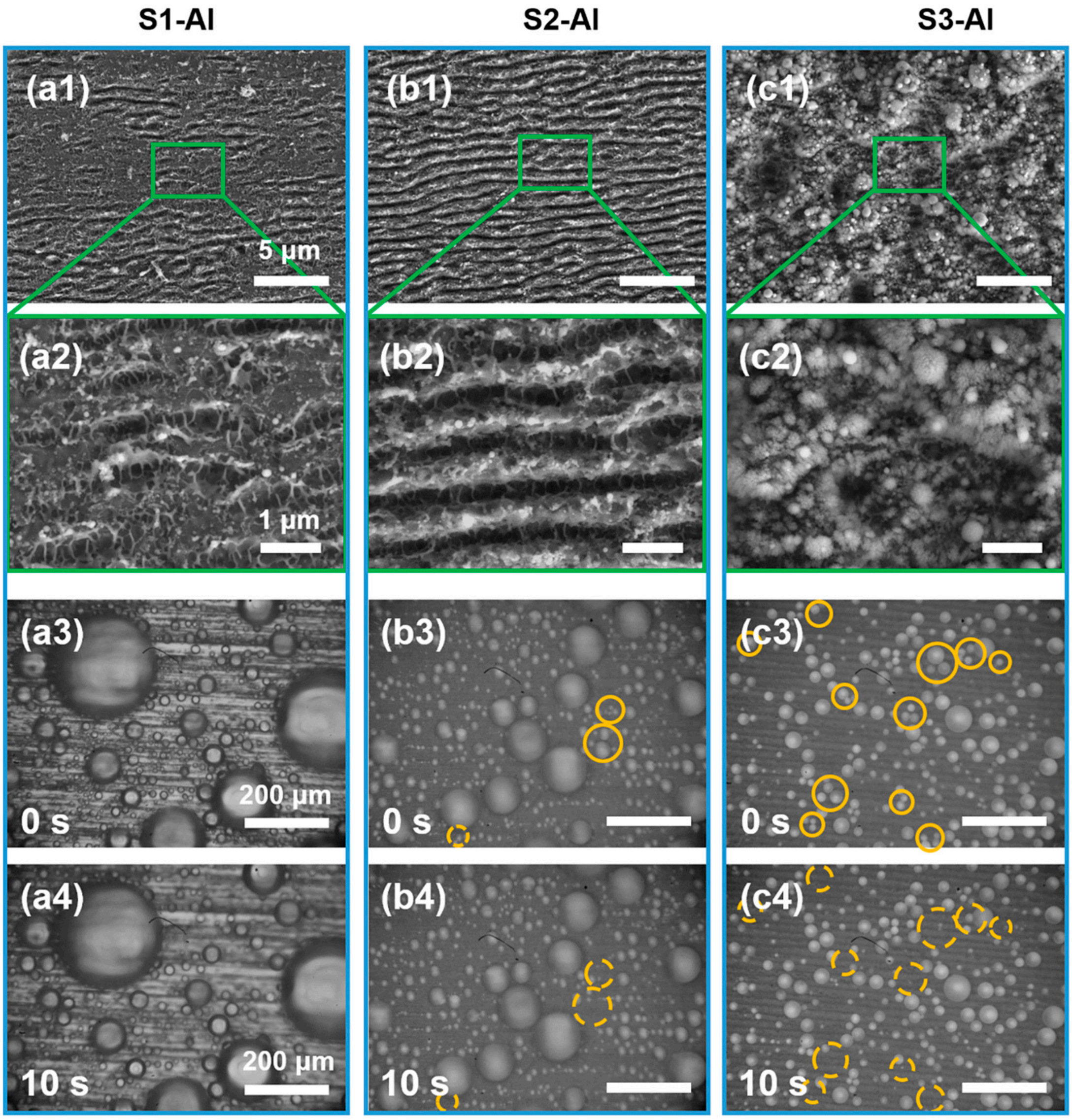

Figure S14. Surface morphology of the ultrafast laser fabricated Al samples at different magnifications and their respective droplet-surface interactions within a 10 seconds interval. (**a**) S1-Al, with little surface structure formation, strong adhesion was induced between the condensed droplets and the surface due to complete wetting in the Wenzel state. No jumping event occurred, and a high amount of accumulated water was present. (**b**) S2-Al, with increased amount of surface nanostructures and reduced adhesion at the solid-liquid interface, scarce jumping events of the condensed droplets were observed, which reduced accumulated water amount as compared to S1-Al. (**c**) S3-Al, abundant nanostructures led to CB state wetting of the condensed droplets and efficient droplet jumping, which resulted in minimized water accumulation.

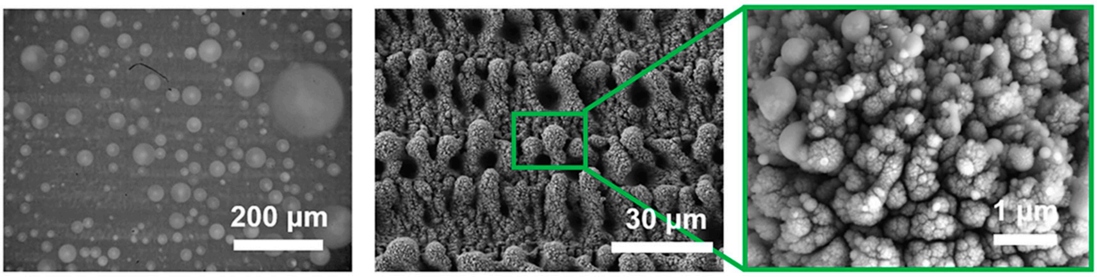

Figure S15. High laser fluence resulted in severe melting of the Al surface due to insufficient heat dissipation. Droplet jumping frequency was comparable to S3-Al, but the amount of accumulated water was increased due to more surface area for condensed droplets to nucleate and grow.

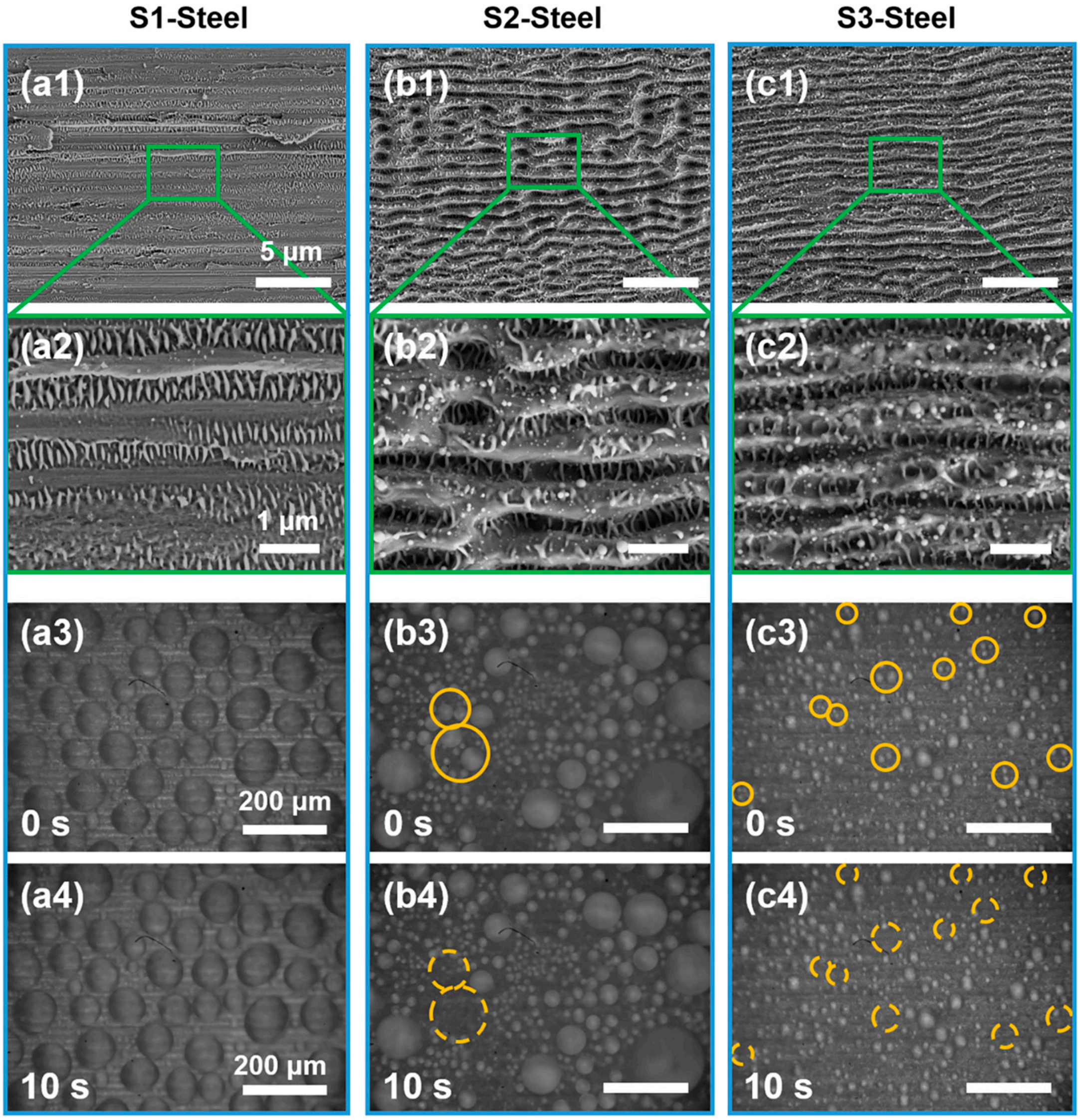

Figure S16. Surface morphology of the ultrafast laser fabricated Steel samples at different magnifications and their respective droplet-surface interactions within a 10 seconds interval. (**a**) S1-Steel, with insufficient surface nanostructuring, there presented Wenzel state wetting and strong adhesion between the condensates and the surface. The condensed droplets adhered to the surface strongly, and no jumping event was observed, which resulted in a large amount of accumulated water. (**b**) S2-Steel, with increased laser fluence, LIPSS could be obtained on the Steel surface. Such surface type exhibited reduced adhesion at the solid-liquid interface and was ascribed to partial-

wetting state instead of Wenzel state. However, there were still only a few droplet jumping events and a large amount accumulated water, which should be unfavorable in antifrosting. (**c**) S3-Steel, the size of the LIPSS reduced, and there was a formation of nanofeatures with size scale suitable for droplet jumping. The combined effect would lead to a decrease in adhesion at the solid-liquid interface, which promoted removal of the condensed droplets and reduced water accumulation.

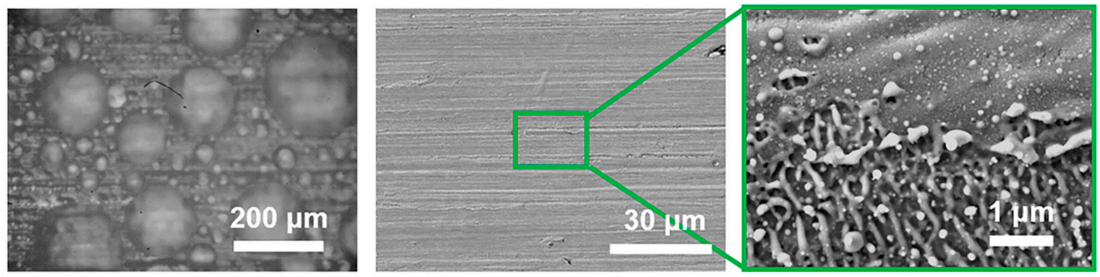

Figure S17. High laser fluence resulted in severe melting of the Steel surface due to insufficient heat dissipation. Differently from Cu and Al, it appears that parts of the irradiated region become nearly flat, possibly due to a lower melting threshold of Steel. As a result, droplet jumping was completely inhibited and coalesced microdroplets would grow large without being removed from the surface over time.

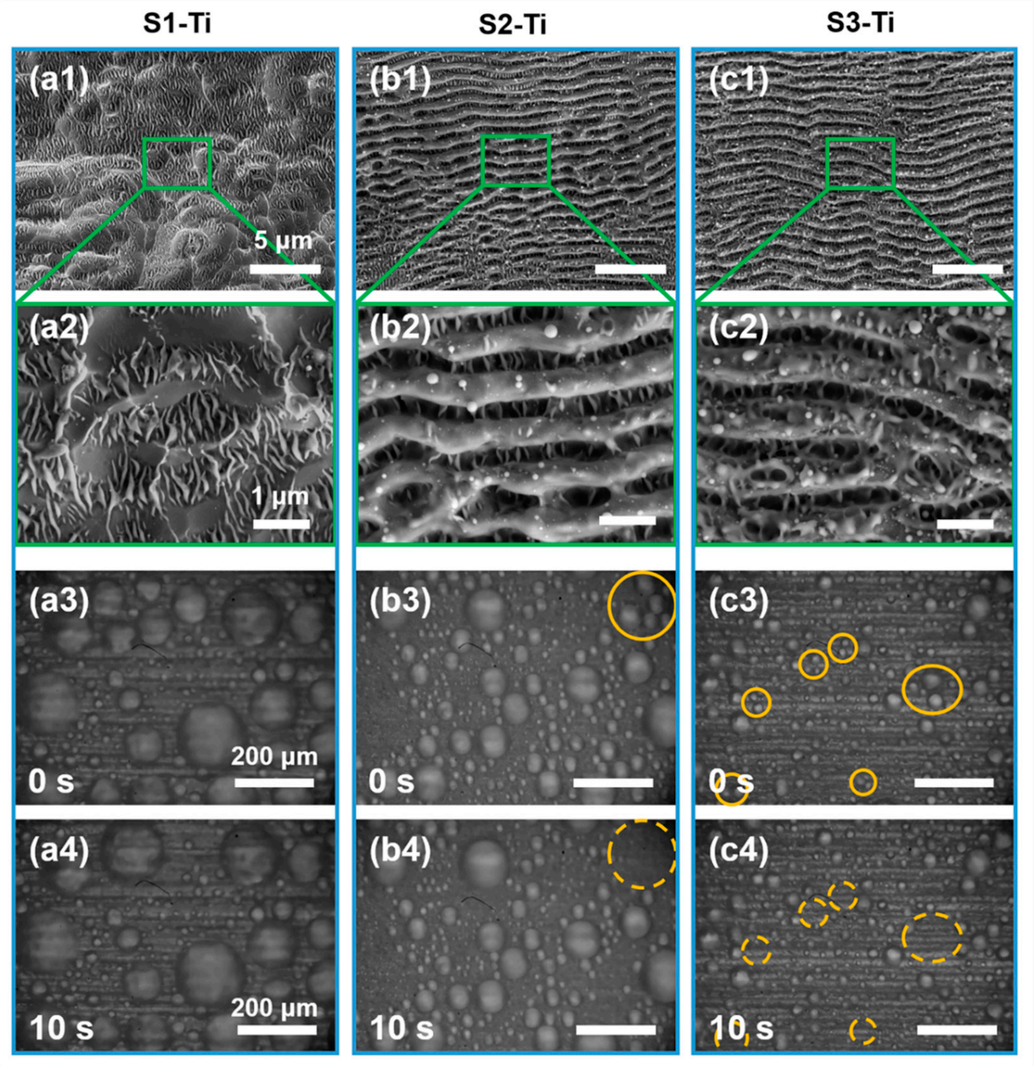

Figure S18. Surface morphology of the ultrafast laser fabricated Ti samples at different magnifications and their respective droplet-surface interactions within a 10 seconds interval. It can be observed that the surface morphology and droplet-surface interaction of Ti is similar to Steel. (**a**) S1-Ti, insufficient surface nanostructuring led to Wenzel state wetting and strong adhesion between the condensates and the surface. No droplet jumping event was recorded, and a large amount of condensed water was accumulated. (**b**) S2-Ti, increase in laser fluence led to the formation of LIPSS, which reduced adhesion at the solid-liquid interface. Only a few droplet jumping events occurred and a large amount of water was still accumulated. (**c**) S3-Ti, with the reduction in the size of LIPSS and the formation of nanostructures, adhesion at the solid-liquid interface was further decreased as compared to S2-Ti, which resulted in efficient removal of the condensed droplets and reduced water accumulation.

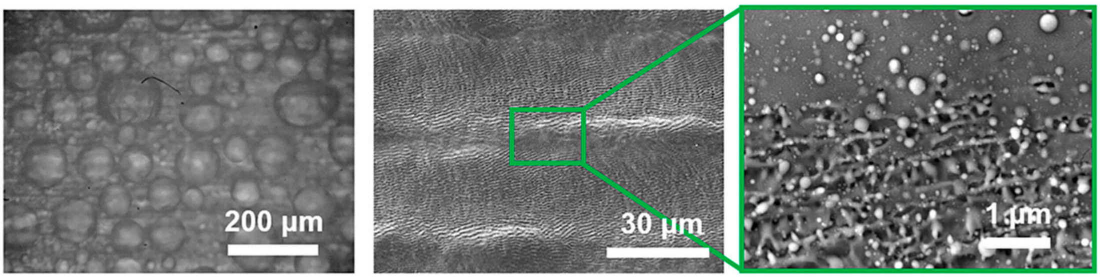

Figure S19. High laser fluence resulted in severe melting of the Ti surface due to insufficient heat dissipation. Similar to the Steel surface, parts of the irradiated region became nearly flat due to melting of Ti rather than ablation. Coalesced droplets would adhere in the Wenzel state without coalesced jumping, and a large amount of accumulated water would present as a result.

SUPPORTING REFERENCES

1. Boreyko, J. B.; Chen, C.-H., Self-Propelled Dropwise Condensate on Superhydrophobic Surfaces. *Physical Review Letters* **2009,** *103* (18), 184501.
2. Yan, X.; Zhang, L.; Sett, S.; Feng, L.; Zhao, C.; Huang, Z.; Vahabi, H.; Kota, A. K.; Chen, F.; Miljkovic, N., Droplet Jumping: Effects of Droplet Size, Surface Structure, Pinning, and Liquid Properties. *ACS Nano* **2019,** *13* (2), 1309-1323.
3. Wang, K.; Liang, Q.; Jiang, R.; Zheng, Y.; Lan, Z.; Ma, X., Self-enhancement of droplet jumping velocity: the interaction of liquid bridge and surface texture. *RSC Advances* **2016,** *6* (101), 99314-99321.
4. Gao, L.; McCarthy, T. J., Wetting 101°. *Langmuir* **2009,** *25* (24), 14105-14115.
5. Seo, D.; Shim, J.; Moon, B.; Lee, K.; Lee, J.; Lee, C.; Nam, Y., Passive Anti-Flooding Superhydrophobic Surfaces. *ACS Applied Materials & Interfaces* **2020,** *12* (3), 4068-4080.
6. Yan, X.; Chen, F.; Sett, S.; Chavan, S.; Li, H.; Feng, L.; Li, L.; Zhao, F.; Zhao, C.; Huang, Z.; Miljkovic, N., Hierarchical Condensation. *ACS Nano* **2019,** *13* (7), 8169-8184.
7. Miljkovic, N.; Enright, R.; Wang, E. N., Effect of Droplet Morphology on Growth Dynamics and Heat Transfer during Condensation on Superhydrophobic Nanostructured Surfaces. *ACS Nano* **2012,** *6* (2), 1776-1785.
